# Near-field and far-field exposures to radiofrequency electromagnetic fields and cancer risks in humans: a protocol for an umbrella review of epidemiological studies

**DOI:** 10.1186/s13643-026-03142-9

**Published:** 2026-03-12

**Authors:** Joshua Louis Ziegler, Susanna Lagorio, Mats-Olof Mattsson, Olga Zeni, John Bolte, Maryse Ledent, Maarten Velghe, Rianne Stam, Kelly Rijs, Isabelle Deltour, Roya Dolatkhah, Daniel Wollschläger, Nikolaos Petroulakis, Lorena Cascant Ortolano, Dan Baaken

**Affiliations:** 1 University Medical Center of the Johannes Gutenberg University Mainz, Institute for Medical Biostatistics, Epidemiology and Informatics (IMBEI), Langenbeckstr. 1, Mainz, 55131 Germany; 2 National Institute of Health (Istituto Superiore di Sanità), Rome, Italy; 3 SciProof International, Östersund, Sweden; 4 National Research Council of Italy (CNR), Institute for Electromagnetic Sensing of the Environment (IREA), Naples, Italy; 5 The Hague University of Applied Sciences, Hague, Netherlands; 6 National Institute for Public Health and the Environment (RIVM), Bilthoven, The Netherlands; 7 Sciensano, Brussels, Belgium; 8 International Agency for Research on Cancer (IARC), Lyon, France; 9 Institute of Computer Science, Foundation for Research and Technology—Hellas (FORTH), Crete, Greece; 10 University Medical Library of Johannes Gutenberg-University, Mainz, Germany; 11 German Federal Office for Radiation Protection (BfS), Competence Center for Electromagnetic Fields (KEMF), Cottbus, Germany

**Keywords:** Umbrella review, Radiofrequency electromagnetic fields, Cancer, Human observational studies, Epidemiology

## Abstract

**Background:**

Exposure to radiofrequency electromagnetic fields (RF-EMF; frequencies 100 kHz to 300 GHz) is ubiquitous. As the use of RF-EMF has grown steadily since the 1950s due to advances in telecommunications and other technologies, concerns about potential health effects have persisted. The World Health Organization (WHO) identified key areas of concern, with cancer being most frequently rated as critical. To synthesize evidence on the association between RF-EMF exposure and neoplastic diseases, we will carry out two separate umbrella reviews to account for different RF-EMF exposure conditions: one will focus on near-field exposure and the other on far-field exposure. Both umbrella reviews will include RF-EMF exposure in living and occupational settings.

**Methods:**

Systematic reviews and meta-analyses of human observational studies on RF-EMF and cancer were searched in MEDLINE, Web of Science Core Collection, EMF-Portal, and Epistemonikos databases. Eligibility criteria will follow the PECOS (Population, Exposure, Comparator, Outcome, Study type) scheme. Eligibility and quality of the identified articles will be evaluated by two reviewers independently. The tools AMSTAR 2 (A MeaSurement Tool to Assess systematic Reviews) and ROBIS (Risk of Bias in Systematic Reviews) will be used to systematically assess methodological quality and risk of bias. Data will be extracted and summarized in a qualitative synthesis using standardized forms and presented in text and tables.

**Discussion:**

These umbrella reviews aim to offer a comprehensive overview of the topic by including systematic reviews and meta-analyses that studied cancer-related health effects of near-field and far-field RF-EMF exposure. In addition, a risk of bias rating will be performed to assess the quality of existing systematic reviews and meta-analyses in the field.

**Systematic review registration:**

PROSPERO CRD42024529007

**Supplementary Information:**

The online version contains supplementary material available at 10.1186/s13643-026-03142-9.

## Background

Exposure to radiofrequency electromagnetic fields (RF-EMF; frequencies 100 kHz to 300 GHz) is ubiquitous. The use of RF-EMF has grown steadily since the 1950s and includes various applications in medicine, industry, domestic appliances, security, military activity, navigation, and especially telecommunications [1].

Since the late 1990s and early 2000s, when mobile telephony became prevalent among the general public, there have been concerns among citizens, governments, and experts about potential health effects of this technology. With the advent of new technological developments in this field, including 5G mobile networks, and increasing wireless connectivity of devices, these concerns remain relevant. Therefore, conducting an evidence-based health risk assessment regarding RF-EMF is crucial to advise decision-makers and to address concerns among the general public.

In 2018, the World Health Organization (WHO) conducted a comprehensive global survey, targeting scientists in the field of RF-EMF health effect research to ascertain the most relevant health effects potentially associated with RF-EMF. Based on the survey findings, six key areas of concern were identified, and the WHO commissioned a series of systematic reviews of observational and experimental studies on the following topics: cancer, adverse reproductive outcomes, cognitive impairment, human self-reported symptoms, oxidative stress, heat-related effects, tinnitus, migraine, and non-specific physical symptoms. Based mainly on evidence from human studies and public concern, cancer was most frequently rated as critical [2].

At exposure levels below limits defined by the International Commission on Non-Ionizing Radiation Protection (ICNIRP), no mechanism for carcinogenicity of RF-EMF is established. Several hundred experimental studies investigated the capacity of RF-EMF radiation to induce genetic damage since genotoxicity is one of the key biological indicators of carcinogenicity, and the most common characteristic of established carcinogens [3, 4]. The results of these studies are inconsistent, with some experimental studies showing significant genotoxicity while others indicate an absence of an effect from RF-EMF exposure. Reported effects are a function of frequency, amplitude, and modulation, but are in most cases not reproduced in follow-up studies [5–8]. Low methodological quality of the initial studies is considered to be the main reason for non-reproducibility [9].

To inform the general public and support policy-makers and healthcare professionals in making sound decisions, all relevant and available scientific evidence should be used. So far, however, no epidemiological study has investigated the risk of neoplastic diseases in relation to individual exposure to RF-EMF from all exposure sources and settings [10–12]. In the face of many, possibly conflicting, primary studies, systematic reviews can provide a comprehensive overview based on a-priori defined methods regarding the inclusion and exclusion of primary studies and evaluate the quality of the body of evidence. As such, systematic reviews are a good evidence synthesis tool and crucial for evidence-based decision-making [13].

A surge of systematic reviews published over the past two decades bears testament to the strong demand for this form of evidence synthesis [14]. However, concerns have been raised regarding the potential susceptibility to bias in systematic reviews and meta-analyses, and the massive production of conflicting, redundant, low-quality and potentially misleading evidence synthesis articles [15]. These concerns also apply to the topic of the association between RF-EMF and cancer. Ioannidis [15] reported on 12 meta-analyses published between 2006 and 2014 with conclusions varying from some potentially increased risk for long-term mobile phone use (≥ 10 years) and ipsilateral gliomas to the interpretation of the results as consistent with a null effect.

In order to synthesize and evaluate the complete body of evidence, it is necessary to conduct overviews of systematic reviews and meta-analyses, so-called umbrella reviews, as scientists have recently started to do in other fields [13, 16, 17]. Umbrella reviews can provide an overview of the complete body of evidence on a certain topic. Because umbrella reviews are conducted using an equally systematic approach as systematic reviews, the results are based on a stronger foundation than common overview articles based on an ad-hoc elective approach. In addition, quantitative approaches, such as those used in the AMSTAR systematic review quality assessment tool (A MeaSurement Tool to Assess systematic Reviews), can be used in umbrella reviews to grade the reliability of evidence on a certain topic [18].

### Objective of the article

The main scope of the reviews is to synthesise all available evidence from published peer-reviewed systematic reviews and meta-analyses on a possible association between RF-EMF exposure and risk of neoplastic diseases in human observational studies, taking different exposure conditions into account. To this end, two separate umbrella reviews (UR-A, UR-B), with UR-A focusing on near-field exposure and UR-B on far-field exposure to RF-EMF, will be carried out. In this article, a two-branched study protocol on UR-A and UR-B is presented.

## Methods/design

This protocol mostly follows the Joanna Briggs Institute (JBI) methodology for umbrella reviews [14, 19]. It deviates from the JBI approach regarding the critical appraisal of the included studies. The protocol is also aligned with the Preferred Reporting Items for Systematic Reviews and Meta-Analysis Protocols 2015 statement (PRISMA-P) [20]. Instead of the critical appraisal checklist for systematic and research synthesis proposed by Aromataris et al. [14, 19], we will use AMSTAR 2 [21] and Risk of Bias in Systematic Reviews (ROBIS) [22], both popular instruments for critically appraising systematic reviews.

### Eligibility criteria

Studies will be included based on the following eligibility criteria as presented in a PECOS (Population Exposure, Comparator, Outcome, Study type) scheme.

#### Population

Both umbrella reviews will include members of the general population and occupationally active individuals. No restrictions on sex, age, or other individual characteristics will be applied.

#### Exposure

In UR-A near-field head-localised RF-EMF exposure from personal or occupational use of wireless devices (mobile phones, cordless phones, hand-held transceivers) or from medical devices such as cochlear implants occurring prior to the outcome event will be examined. In UR-B far-field whole body environmental or occupational RF-EMF exposure from radio-television transmitters, base stations or any other fixed-site transmitter, occurring prior to the outcome event will be investigated.

Both umbrella reviews depend on the exposure variables and metrics most commonly used in the published systematic reviews and meta-analyses, namely indirect measures. Some examples are mobile phone carrier subscriber data, self-reported usage, and measurements or modelled levels of electric fields, magnetic fields or (incident) power density, e.g., at the subject’s residence. Systematic reviews and meta-analyses that included occupational RF-EMF exposure assessed, e.g., via personal exposure measurements, Job Exposure Matrices, or job titles will also be considered, provided that the assessment of the impact of RF-EMF exposure was a predefined research objective and that the exposure is well characterised in terms of source and type.

#### Comparator

The eligible systematic reviews or meta-analyses should include studies appropriate to provide estimates of the relative risk of diseases based on exposure, i.e., comparing a group of unexposed or less exposed individuals with exposed or highly exposed individuals.

#### Outcomes

The umbrella reviews will include all types of neoplasms reported in published systematic reviews and meta-analyses on exposure to RF-EMF that are either histologically confirmed or based on unequivocal diagnostic imaging or ascertainment through cancer registries, hospitals, or any other source that has a sufficient coverage of the study base during the time of the study. Self-reported outcomes will also be included if they are included in a systematic review or meta-analysis identified in our umbrella reviews. Systematic reviews and meta-analyses eligible for inclusion in the umbrella reviews will be based preferably on studies investigating incident tumour cases. However, systematic reviews and meta-analyses based on studies analysing cancer mortality will not be excluded.

#### Study type

Systematic reviews and meta-analyses of epidemiological studies will be eligible for inclusion if they are informative for hazard assessment purposes, i.e., they summarised the evidence provided by aetiological studies of cohort or case-control design (including variants thereof) published in peer-reviewed journals. Non-systematic reviews such as narrative reviews, scoping reviews or evidence maps will not be included. Primary level studies will not be included, regardless of their design (such as ecological studies, cross-sectional studies, case–case analyses, case-control studies, cohort studies and nested case-control studies). Pooled studies of, e.g., case-control or cohort studies will be considered as primary level studies and therefore will not be included.

### Search strategy

The following electronic databases were searched from inception to 15 May 2024 for eligible studies: MEDLINE (PubMed), Web of Science Core Collection (Clarivate), EMF-Portal, and Epistemonikos. The search strategy was structured to represent the PECOS schematic of this review. We imposed no restrictions on language or publication date on any of the searches. For our MEDLINE search we added a filter for identifying Systematic Reviews and Meta-analysis [23]. The approach to study identification from this umbrella review is transparently reported in the Additional file 1.

Furthermore, a manual search of all reference lists of the included articles will be performed to identify additional systematic reviews of relevance. Relevant articles found via a manual search of the author’s personal libraries will also be included. However, unpublished articles will not be sought.

Searches will be re-run prior to the final analysis and additional relevant systematic reviews or meta-analyses identified will be included. A final search will be performed in EMF-Portal shortly before the results are published.

### Study screening

The EndNote 21 software (https://endnote.com) is used for assembling results of the literature search and further data management during the process. For duplicate removal, the Deduplicator tool of The Systematic Review Accelerator [24] was used in combination with a self-written R script (Additional file 5). Two team members (DB, JZ) will independently evaluate the eligibility of identified articles for inclusion using RAYYAN [25], first screening the title and abstract and later assessing the full text of those articles which passed the first step of screening. If no consensus is achieved, a third reviewer will be included.

### Data extraction

Data will be extracted independently by two reviewers using pre-designed and pre-piloted forms (Additional file 2). The tool may be modified if issues arise during testing. These standardised abstraction forms will be established in Microsoft Excel. Ambiguities related to data extraction will be resolved by discussion or by a third reviewer if the two primary reviewers are unable to achieve consensus. When necessary, if data is not provided in the article, we will contact the corresponding authors to provide the missing data.

### Data synthesis

The data from the included studies will be summarised in a qualitative synthesis of the findings using text and tables describing study characteristics and the overall results of the umbrella review. The results will be presented separately for UR-A and UR-B. We will also present the studies in each umbrella review (UR-A and UR-B) stratified by exposure to the general population and occupational exposure if the included systematic reviews and meta-analyses allow for this. Primary research study level data will not be presented in the umbrella review, which is in line with the JBI approach [19].

A clear indication of overlap between primary studies in each of the included systematic reviews and meta-analyses will be presented in the umbrella review. If one study is included multiple times, this will be pointed out.

### Critical appraisal of identified studies

To assess the methodological quality and risk of bias of the included systematic reviews and meta-analyses in these umbrella reviews, AMSTAR 2 [21] and ROBIS [22] will be used (Additional files 3 and 4). The critical appraisal will be performed independently by two reviewers. Conflicts in the rating of the studies will be resolved by consensus. If no consensus between the two reviewers can be achieved, the conflict will be resolved by arbitration by a third reviewer of the team. The initial individual ratings and the final modified rating after resolving potential conflicts will be recorded. Review authors will not extract data or assess risk of bias of any study on which they were an author.

## Discussion

Exposure to RF-EMF has become widespread, encompassing various sectors like telecommunications, medicine, industry, and more. Concern over potential health effects of mobile communication technology persists, particularly with the regular emergence of new communication protocols, such as the 5th generation communication protocol in the early 2020 s (so-called 5G New Radio). To address these concerns, conducting a health risk assessment for RF-EMF is essential for supporting decisions by policymakers as well as informing the scientific and healthcare community and the public. These umbrella reviews aim to offer a comprehensive overview of the topic by including systematic reviews and meta-analyses that studied cancer-related health effects of near-field and far-field RF-EMF exposure. In addition, a risk of bias rating will be performed to assess the quality of existing systematic reviews and meta-analyses in the field.

## Supplementary Information

Additional file 1: Search strategies for MEDLINE via PubMed, Web of Science, Epistemonikos and EMF-Portal, as performed on May 15th 2024. Description: Detailed depiction of search strategies for MEDLINE (PubMed), Web of Science Core Collection (Clarivate), EMF-Portal, and Epistemonikos that were used to search for relevant articles on May 15th 2024.

Additional file 2: Preliminary data extraction tool. Description: Preliminary data extraction tool that will be used to collect relevant article data.

Additional file 3: AMSTAR 2: a critical appraisal tool for systematic reviews that include randomised or nonrandomised studies of healthcare interventions, or both. Description: A Microsoft Word sheet that will be used for applying the critical appraisal criteria of AMSTAR 2.

Additional file 4: ROBIS: Tool to assess risk of bias in systematic reviews. Description: A Microsoft Word sheet that will be used for applying the critical appraisal criteria of ROBIS.

Additional file 5: R script for removing (fuzzy) duplicates from bibliography. Description: This R script checks for fuzzy and exact duplicates in the bibliography and was used in addition to other deduplication tools.

## Data Availability

Not applicable.

## Abbreviations

AMSTAR: A MeaSurement Tool to Assess systematic Reviews
ICNIRP: International Commission on Non-Ionizing Radiation Protection
JBI: Joanna Briggs Institute
PECOS: Population, Exposure, Comparator, Outcome, Study type
RF-EMF: Radiofrequency electromagnetic fields
ROBIS: Risk of Bias in Systematic Reviews
UR-A: Umbrella review concerning near-field exposure to RF-EMF
UR-B: Umbrella review concerning far-field exposure to RF-EMF
WHO: World Health Organization
