# Supplementary material for: Near-field and far-field exposures to radiofrequency electromagnetic fields and cancer risks in humans: a protocol for an umbrella review of epidemiological studies
Source: Syst Rev. 2026 Mar 12;15:130. doi: 10.1186/s13643-026-03142-9 (PMC13072611; doi:10.1186/s13643-026-03142-9)
Supplement: Supplementary file 1 — Additional file 1: Search strategies for MEDLINE via PubMed, Web of Science, Epistemonikos and EMF-Portal, as performed on May 15th 2024. Description: Detailed depiction of search strategies for MEDLINE (PubMed), Web of Science Core Collection (Clarivate), EMF-Portal, and Epistemonikos that were used to search for relevant articles on May 15th 2024. [file 13643_2026_3142_MOESM1_ESM.docx]

**Additional file 1: Search strategies for MEDLINE via PubMed, Web of Science, Epistemonikos and EMF-Portal, as performed on May 15th 2024**

Table 1: Search strategies from MEDLINE via PubMed

| **#** | **Search Term** | **Results** |
| --- | --- | --- |
| 1 | "Electromagnetic Fields"[MeSH Terms] OR "RF-EMF"[Title/Abstract] OR "radiofrequency electromagnetic field*"[Title/Abstract] OR "radiofrequency field*"[Title/Abstract] OR "radio frequency field*"[Title/Abstract] OR "Far-field"[Title/Abstract] OR "near-field"[Title/Abstract] OR "Wireless Technology"[MeSH Terms] OR "Cell Phone"[MeSH Terms] OR "Cell Phone Use"[MeSH Terms] OR "Radio"[MeSH Terms] OR "mobile telephone*"[Title/Abstract] OR "smartphone*"[Title/Abstract] OR "smart phone*"[Title/Abstract] OR "cell phone*"[Title/Abstract] OR "mobile phone*"[Title/Abstract] OR "cellular phone*"[Title/Abstract] OR "cellular telephone*"[Title/Abstract] OR "Radio Waves"[MeSH Terms] OR "broadcasting"[Title/Abstract] OR "radiation, nonionizing"[MeSH Terms:noexp] OR "nonionizing radiation*"[Title/Abstract] OR "non ionizing radiation*"[Title/Abstract] OR "radio wave*"[Title/Abstract] OR "radiowave*"[Title/Abstract] OR "hertzian wave*"[Title/Abstract] OR "high frequency wave*"[Title/Abstract] OR "short wave*"[Title/Abstract] OR "microwave*"[Title/Abstract] OR "micro wave*"[Title/Abstract] OR "ultrahigh frequency wave*"[Title/Abstract] OR "ehf wave*"[Title/Abstract] | 154,475 |
| 2 | "neoplasms"[MeSH Terms] OR "adenosarcoma*"[Title/Abstract] OR "blastoma*"[Title/Abstract] OR "cancer*"[Title/Abstract] OR "carcinoma*"[Title/Abstract] OR "carcinosarcoma*"[Title/Abstract] OR "glioma*"[Title/Abstract] OR "leukemia*"[Title/Abstract] OR "leukaemia*"[Title/Abstract] OR "liposarcoma*"[Title/Abstract] OR "lymphangiom*"[Title/Abstract] OR "lymphoma*"[Title/Abstract] OR "melanoma*"[Title/Abstract] OR "metastatic"[Title/Abstract] OR "myeloma*"[Title/Abstract] OR "neoplasm*"[Title/Abstract] OR "osteosarcoma*"[Title/Abstract] OR "sarcoma*"[Title/Abstract] OR "tumor*"[Title/Abstract] OR "tumour*"[Title/Abstract] OR "meningioma*"[Title/Abstract] OR "acoustic neuroma*"[Title/Abstract] OR "vestibular schwannoma"[Title/Abstract] | 5,281,013 |
| 3 | #1 and #2 | 14,189 |
| 4 | ("plants"[MeSH Terms] OR "animals"[MeSH Terms]) NOT "humans"[MeSH Terms] | 5,408,761 |
| 5 | #3 not #4 | 12,983 |
| 6 | (("Systematic Review"[Publication Type:noexp] OR "Systematic Reviews as Topic"[MeSH Terms:noexp] OR "Cochrane Database Syst Rev"[Journal] OR "evid rep technol assess full rep"[Journal] OR "evid rep technol assess summ"[Journal] OR "scoping"[Title] OR "systematic"[Title] OR ((("comprehensive analysis"[Title/Abstract:~1] OR "comprehensive review"[Title/Abstract:~1] OR "comprehensively reviewed"[Title/Abstract:~1] OR "literature search"[Title/Abstract:~1] OR "literature searches"[Title/Abstract:~1] OR "scoping search"[Title/Abstract:~1] OR "scoping searches"[Title/Abstract:~1]) NOT "narrative review"[Title]) OR "pooled study"[Title/Abstract:~1] OR "systematic search"[Title/Abstract:~1] OR "systematic searches"[Title/Abstract:~1] OR "systematically searched"[Title/Abstract:~1])) AND ("databases"[Title/Abstract] OR "cinahl"[Title/Abstract] OR "cochrane"[Title/Abstract] OR "embase"[Title/Abstract] OR "psycinfo"[Title/Abstract] OR "pubmed"[Title/Abstract] OR "medline"[Title/Abstract] OR "scopus"[Title/Abstract] OR "web science"[Title/Abstract:~1] OR "bibliographic review"[Title/Abstract:~1] OR "bibliographic reviews"[Title/Abstract:~1] OR "literature review"[Title/Abstract:~1] OR "literature reviews"[Title/Abstract:~1])) OR (("electronic database"[Title/Abstract:~1] OR "electronic databases"[Title/Abstract:~1] OR "databases searched"[Title/Abstract:~3]) AND ("eligibility"[Title/Abstract] OR "excluded"[Title/Abstract] OR "exclusion"[Title/Abstract] OR "included"[Title/Abstract] OR "inclusion"[Title/Abstract])) OR ("comparative effectiveness"[Title/Abstract:~1] AND "effectiveness review"[Title/Abstract:~2]) OR ("critical interpretive"[Title/Abstract:~1] AND ("interpretive review"[Title/Abstract:~0] OR "interpretive synthesis"[Title/Abstract:~0])) OR ("diagnostic test"[Title/Abstract:~0] AND ("accuracy review"[Title/Abstract] OR "accuracy reviews"[Title/Abstract] OR "accuracy studies"[Title/Abstract] OR "accuracy study"[Title/Abstract]) AND ("meta analysis"[Title/Abstract] OR "scoping"[Title/Abstract] OR "systematic"[Title/Abstract])) OR ("evidence assessment"[Title/Abstract] AND "GRADE"[Title/Abstract]) OR ("evidence gap"[Title/Abstract:~2] AND "gap map"[Title/Abstract:~0]) OR "evidence mapping"[Title/Abstract] OR "evidence review"[Title/Abstract] OR "exploratory review"[Title/Abstract] OR "framework synthesis"[Title/Abstract] OR "mapping review"[Title/Abstract:~1] OR "meta epidemiological"[Title/Abstract] OR "meta ethnographic"[Title/Abstract:~0] OR "metaethnographic"[Title/Abstract] OR "meta ethnography"[Title/Abstract:~0] OR "metaethnography"[Title/Abstract] OR "meta interpretation"[Title/Abstract:~1] OR "meta narrative"[Title/Abstract:~1] OR "meta review"[Title/Abstract:~1] OR "meta study"[Title/Abstract:~1] OR "meta synthesis"[Title/Abstract:~0] OR "metasynthesis"[Title/Abstract] OR "meta summary"[Title/Abstract:~1] OR "meta theory"[Title/Abstract:~1] OR "methodological review"[Title/Abstract:~1] OR "methodology review"[Title/Abstract:~1] OR ("mixed methods"[Title/Abstract:~0] AND "methods review"[Title/Abstract:~1]) OR ("mixed methods"[Title/Abstract:~0] AND "methods synthesis"[Title/Abstract:~1]) OR "narrative synthesis"[Title/Abstract:~1] OR "overview reviews"[Title/Abstract:~4] OR ("PRISMA"[Title/Abstract] AND ("guideline"[Title/Abstract] OR "guidelines"[Title/Abstract] OR "preferred"[Title/Abstract] OR "reporting"[Title/Abstract] OR "requirements"[Title/Abstract])) OR "PRISMA-P"[Title/Abstract:~0] OR "prognostic review"[Title/Abstract:~1] OR "psychometric review"[Title/Abstract:~1] OR ("qualitative evidence"[Title/Abstract:~0] AND "evidence synthesis"[Title/Abstract:~0]) OR ("qualitative research"[Title/Abstract:~0] AND "research synthesis"[Title/Abstract:~0]) OR ("rapid evidence"[Title/Abstract:~0] AND "evidence assessment"[Title/Abstract:~0]) OR "rapid realist"[Title/Abstract:~0] OR "rapid review"[Title/Abstract:~1] OR "rapid reviews"[Title/Abstract:~1] OR "realist review"[Title/Abstract:~1] OR ("review economic"[Title/Abstract:~1] AND ("economic evaluation"[Title/Abstract:~1] OR "economic evaluations"[Title/Abstract:~1])) OR "review reviews"[Title/Abstract:~1] OR "realist syntheses"[Title/Abstract:~1] OR "realist synthesis"[Title/Abstract:~1] OR "scoping review"[Title/Abstract:~2] OR "scoping reviews"[Title/Abstract:~2] OR "scoping studies"[Title/Abstract:~2] OR "scoping study"[Title/Abstract:~2] OR "systematic evidence map"[Title/Abstract] OR "systematic mapping"[Title/Abstract:~2] OR "systematic literature"[Title/Abstract:~1] OR "systematic Medline"[Title/Abstract:~2] OR "systematic PubMed"[Title/Abstract:~2] OR "Systematic Review"[Title/Abstract:~2] OR "systematic reviews"[Title/Abstract:~2] OR "systematical review"[Title/Abstract:~1] OR "systematical reviews"[Title/Abstract:~2] OR "systematically identified"[Title/Abstract:~1] OR "systematically review"[Title/Abstract:~1] OR "systematically reviewed"[Title/Abstract:~1] OR "systematized review"[Title/Abstract:~1] OR "umbrella review"[Title/Abstract:~2] OR "umbrella reviews"[Title/Abstract:~2] OR "meta-analysis as topic"[MeSH Terms:noexp] OR "meta analysis"[Publication Type] OR "network meta-analysis"[MeSH Terms:noexp] OR "indirect comparison"[Title/Abstract:~1] OR "meta analyses"[Title/Abstract] OR "meta analysis"[Title/Abstract] OR "meta analytic"[Title/Abstract] OR "meta analytical"[Title/Abstract] OR "meta analytics"[Title/Abstract] OR "meta analyze"[Title/Abstract] OR "meta analyzed"[Title/Abstract] OR "metaanalyses"[Title/Abstract] OR "metaanalysis"[Title/Abstract] OR "metaanalytic"[Title/Abstract] OR "metaanalyze"[Title/Abstract] OR "metaanalyzed"[Title/Abstract] OR "network comparison"[Title/Abstract:~1] OR "network meta analyses"[Title/Abstract] OR "network meta-analysis"[Title/Abstract] OR "network metaanalyses"[Title/Abstract] OR "network metaanalysis"[Title/Abstract] OR ("systematic"[Title/Abstract] AND ("meta regression"[Title/Abstract] OR "metaregression"[Title/Abstract])) | 599,790 |
| 7 | #5 and #6 | 553 |

Table 2: Search strategies from the Web of Science Core Collection

| **#** | **Search Term** | **Results** |
| --- | --- | --- |
| 1 | TS=(RF-EMF) | 647 |
| 2 | TS=((radiofrequency OR radio-frequency) near/3 field*) | 5,988 |
| 3 | TS=(near-field) | 50,965 |
| 4 | TS=(Far-field) | 40,297 |
| 5 | TS=((mobile or cellular) NEAR/3 telephone*) | 2,965 |
| 6 | TS=(smartphone*) | 72,629 |
| 7 | TS=((smart OR cell OR mobile OR Cellular) NEAR/3 phone*) | 61,629 |
| 8 | TS=(broadcasting) | 61,806 |
| 9 | TS=((nonionizing OR ""non ionizing"") NEAR/3 radiation*) | 2,032 |
| 10 | TS=(radiowave*) | 2,016 |
| 11 | TS=((hertzian or radio or ""high frequency"" or ""short"" or micro or ehf or ""ultrahigh frequency"") near/3 wave*) | 61,754 |
| 12 | TS=(microwave*) | 328,031 |
| 13 | #12 OR #11 OR #10 OR #9 OR #8 OR #7 OR #6 OR #5 OR #4 OR #3 OR #2 OR #1 | 657,287 |
| 14 | TS=(adenosarcoma* OR blastoma* OR cancer* OR carcinoma* OR carcinosarcoma* OR glioma* OR leukemia* OR leukaemia* OR liposarcoma* OR lymphangiom* OR lymphoma* OR melanoma* OR metastatic OR myeloma* OR neoplasm* OR osteosarcoma* OR sarcoma* OR tumor* OR tumour* OR meningioma* OR ""acoustic neuroma*"" OR ""vestibular schwannoma"") | 5,468,746 |
| 15 | #13 AND #14 | 17,543 |
| 16 | #13 AND #14 and Review Article (Document Types) | 2,034 |

Table 3: Search strategies from Epistemonikos

| **#** | **Search Term** | **Results** |
| --- | --- | --- |
|  | (title:(adenosarcoma* OR blastoma* OR cancer* OR carcinoma* OR carcinosarcoma* OR glioma* OR leukemia* OR leukaemia* OR liposarcoma* OR lymphangiom* OR lymphoma* OR melanoma* OR metastatic OR myeloma* OR neoplasm* OR osteosarcoma* OR sarcoma* OR tumor* OR tumour* OR meningioma* OR "acoustic neuroma" OR "vestibular schwannoma") OR abstract:(adenosarcoma* OR blastoma* OR cancer* OR carcinoma* OR carcinosarcoma* OR glioma* OR leukemia* OR leukaemia* OR liposarcoma* OR lymphangiom* OR lymphoma* OR melanoma* OR metastatic OR myeloma* OR neoplasm* OR osteosarcoma* OR sarcoma* OR tumor* OR tumour* OR meningioma* OR "acoustic neuroma" OR "vestibular schwannoma")) AND (title:("RF-EMF" OR "radiofrequency electromagnetic field" OR "radiofrequency field" OR "radio frequency field" OR "Far-field" OR "near-field" OR "mobile telephone" OR smartphone* OR "smart phone" OR "cell phone" OR "mobile phone" OR "cellular phone" OR "cellular telephone" OR broadcasting OR "nonionizing radiation" OR "non ionizing radiation" OR "radio wave" OR "radiowave" OR "hertzian wave" OR "high frequency wave" OR "short wave" OR microwave* OR "micro wave" OR "ultrahigh frequency wave" OR "ehf wave" OR "radiofrequency electromagnetic fields" OR "radiofrequency fields" OR "radio frequency fields" OR "near-fields" OR "Far-fields" OR "mobile telephones" OR smartphone* OR "smart phones" OR "cell phones" OR "mobile phones" OR "cellular phones" OR "cellular telephones" OR broadcasting OR "nonionizing radiations" OR "non ionizing radiations" OR "radio waves" OR "radiowaves" OR "hertzian waves" OR "high frequency waves" OR "short waves" OR microwave* OR "micro waves" OR "ultrahigh frequency waves" OR "ehf waves") OR abstract:("RF-EMF" OR "radiofrequency electromagnetic field" OR "radiofrequency field" OR "radio frequency field" OR "Far-field" OR "near-field" OR "mobile telephone" OR smartphone* OR "smart phone" OR "cell phone" OR "mobile phone" OR "cellular phone" OR "cellular telephone" OR broadcasting OR "nonionizing radiation" OR "non ionizing radiation" OR "radio wave" OR "radiowave" OR "hertzian wave" OR "high frequency wave" OR "short wave" OR microwave* OR "micro wave" OR "ultrahigh frequency wave" OR "ehf wave" OR "radiofrequency electromagnetic fields" OR "radiofrequency fields" OR "radio frequency fields" OR "near-fields" OR "Far-fields" OR "mobile telephones" OR smartphone* OR "smart phones" OR "cell phones" OR "mobile phones" OR "cellular phones" OR "cellular telephones" OR broadcasting OR "nonionizing radiations" OR "non ionizing radiations" OR "radio waves" OR "radiowaves" OR "hertzian waves" OR "high frequency waves" OR "short waves" OR microwave* OR "micro waves" OR "ultrahigh frequency waves" OR "ehf waves")) | 2,095 |
|  | Filter: Publication type = Systematic Review | 305 |

Table 4: Search strategies from EMF-Portal

| **#** | **Search Term** | **Results** |
| --- | --- | --- |
|  | cancer OR tumour AND (topic=epidemiological OR topic=review_survey_summary) AND (frequencyRange=radio_frequency OR frequencyRange=mobile_communications) | 529 |
