# Supplementary material for: Near-field and far-field exposures to radiofrequency electromagnetic fields and cancer risks in humans: a protocol for an umbrella review of epidemiological studies
Source: Syst Rev. 2026 Mar 12;15:130. doi: 10.1186/s13643-026-03142-9 (PMC13072611; doi:10.1186/s13643-026-03142-9)
Supplement: Supplementary file 2 — Additional file 2: Preliminary data extraction tool. Description: Preliminary data extraction tool that will be used to collect relevant article data. [file 13643_2026_3142_MOESM2_ESM.docx]

**Additional file 2: Preliminary data extraction tool**

| Citation details |
| --- |
| Objectives |
| Type of review |
| Number of databases sourced and searched |
| Date range of database searching |
| Publication date range of studies included that inform each outcome of interest |
| Number of studies |
| Types of studies |
| Country of origin of studies |
| Details about participants |
| Setting and context of the original studies reviewed |
| Instrument(s) used to appraise the primary studies and rating of their quality |
| Exposure information |
| Outcomes reported that are relevant to the umbrella review research question |
| Method of synthesis/analysis employed to summarise the evidence |
| Funding of the study |
| Comments or notes |
