# Supplementary material for: Near-field and far-field exposures to radiofrequency electromagnetic fields and cancer risks in humans: a protocol for an umbrella review of epidemiological studies
Source: Syst Rev. 2026 Mar 12;15:130. doi: 10.1186/s13643-026-03142-9 (PMC13072611; doi:10.1186/s13643-026-03142-9)
Supplement: Supplementary file 3 — Additional file 3: AMSTAR 2: a critical appraisal tool for systematic reviews that include randomised or nonrandomised studies of healthcare interventions, or both. Description: A Microsoft Word sheet that will be used for applying the critical appraisal criteria of AMSTAR 2. [file 13643_2026_3142_MOESM3_ESM.docx]

**AMSTAR 2: a critical appraisal tool for systematic reviews that include randomised or nonrandomised studies of healthcare interventions, or both**

| **Study ID** | **Study Reference** | **Assessor** | **Date** |
| --- | --- | --- | --- |
|  |  |  |  |

**1. Did the research questions and inclusion criteria for the review include the components of PICO?**

| Yes  No | For Yes:  Population  Intervention  Comparator group  Outcome | Optional (recommended):  Timeframe for follow-up |
| --- | --- | --- |
| **Rating rationale:** | | |

**2. Did the report of the review contain an explicit statement that the review methods were established prior to the conduct of the review and did the report justify any significant deviations from the protocol?**

| Yes  Partial Yes  No | For Partial Yes:  The authors state that they had a written protocol or guide that included ALL the following:  review question(s)  a search strategy  inclusion/exclusion criteria  a risk of bias assessment | For Yes:  As for partial yes, plus the protocol should be registered and should also have specified:  a meta-analysis/synthesis plan, if appropriate, and  a plan for investigating causes of heterogeneity  justification for any deviations from the protocol |
| --- | --- | --- |
| **Rating rationale:** | | |

**3. Did the review authors explain their selection of the study designs for inclusion in the review?**

| Yes  No | For Yes, the review should satisfy ONE of the following:  Explanation for including only RCTs  OR Explanation for including only NRSI  OR Explanation for including both RCTs and NRSI |
| --- | --- |
| **Rating rationale:** | |

**4. Did the review authors use a comprehensive literature search strategy?**

| Yes  Partial Yes  No | For Partial Yes (all the following):  searched at least 2 databases (relevant to research question)  provided key word and/or search strategy  justified publication restrictions (e.g. language) | For Yes, should also have (all the following):  searched the reference lists / bibliographies of included studies  searched trial/study registries  included/consulted content experts in the field  where relevant, searched for grey literature  conducted search within 24 months of completion of the review |
| --- | --- | --- |
| **Rating rationale:** | | |

**5. Did the review authors perform study selection in duplicate?**

| Yes  No | For Yes, either ONE of the following:  at least two reviewers independently agreed on selection of eligible studies and achieved consensus on which studies to include  OR two reviewers selected a sample of eligible studies and achieved good agreement (at least 80 percent), with the remainder selected by one reviewer. |
| --- | --- |
| **Rating rationale:** | |

**6. Did the review authors perform data extraction in duplicate?**

| Yes  No | For Yes, either ONE of the following:  at least two reviewers achieved consensus on which data to extract from included studies  OR two reviewers extracted data from a sample of eligible studies and achieved good agreement (at least 80 percent), with the remainder extracted by one reviewer. |
| --- | --- |
| **Rating rationale:** | |

**7. Did the review authors provide a list of excluded studies and justify the exclusions?**

| Yes  Partial Yes  No | For Partial Yes:  provided a list of all potentially relevant studies that were read in full-text form but excluded from the review | For Yes, must also have:  Justified the exclusion from the review of each potentially relevant study |
| --- | --- | --- |
| **Rating rationale:** | | |

**8. Did the review authors describe the included studies in adequate detail?**

| Yes  Partial Yes  No | For Partial Yes (ALL the following):  described populations  described interventions  described comparators  described outcomes  described research designs | For Yes, should also have ALL the following:  described population in detail  described intervention in detail (including doses where relevant)  described comparator in detail (including doses where relevant)  described study’s setting  timeframe for follow-up |
| --- | --- | --- |
| **Rating rationale:** | | |

**9. Did the review authors use a satisfactory technique for assessing the risk of bias (RoB) in individual studies that were included in the review?**

| **RCTs**  Yes  Partial Yes  No  Includes only NRSI | For Partial Yes, must have assessed RoB from  unconcealed allocation, and  lack of blinding of patients and assessors when assessing outcomes (unnecessary for objective outcomes such as all-cause mortality) | For Yes, must also have assessed RoB from:  allocation sequence that was not truly random, and  selection of the reported result from among multiple measurements or analyses of a specified outcome |
| --- | --- | --- |
| **NRSI**  Yes  Partial Yes  No  Includes only RCTs | For Partial Yes, must have assessed RoB:  from confounding, and  from selection bias | For Yes, must also have assessed RoB:  methods used to ascertain exposures and outcomes, and  selection of the reported result from among multiple measurements or analyses of a specified outcome |
| **Rating rationale:** | | |

**10. Did the review authors report on the sources of funding for the studies included in the review?**

| Yes  No | For Yes  Must have reported on the sources of funding for individual studies included in the review. Note: Reporting that the reviewers looked for this information but it was not reported by study authors also qualifies |
| --- | --- |
| **Rating rationale:** | |

**11. If meta-analysis was performed did the review authors use appropriate methods for statistical combination of results?**

| **RCTs**  Yes  No  No meta-analysis conducted | For Yes:  The authors justified combining the data in a meta-analysis  AND they used an appropriate weighted technique to combine study results and adjusted for heterogeneity if present.  AND investigated the causes of any heterogeneity |
| --- | --- |
| **For NRSI**  Yes  No  No meta-analysis conducted | For Yes:  The authors justified combining the data in a meta-analysis  AND they used an appropriate weighted technique to combine study results, adjusting for heterogeneity if present  AND they statistically combined effect estimates from NRSI that were adjusted for confounding, rather than combining raw data, or justified combining raw data when adjusted effect estimates were not available  AND they reported separate summary estimates for RCTs and NRSI separately when both were included in the review |
| **Rating rationale:** | |

**12. If meta-analysis was performed, did the review authors assess the potential impact of RoB in individual studies on the results of the meta-analysis or other evidence synthesis?**

| Yes  No  No meta-analysis conducted | For Yes:  included only low risk of bias RCTs  OR, if the pooled estimate was based on RCTs and/or NRSI at variable RoB, the authors performed analyses to investigate possible impact of RoB on summary estimates of effect. |
| --- | --- |
| **Rating rationale:** | |

**13. Did the review authors account for RoB in individual studies when interpreting/ discussing the results of the review?**

| Yes  No | For Yes:  included only low risk of bias RCTs  OR, if RCTs with moderate or high RoB, or NRSI were included the review provided a discussion of the likely impact of RoB on the results |
| --- | --- |
| **Rating rationale:** | |

**14. Did the review authors provide a satisfactory explanation for, and discussion of, any heterogeneity observed in the results of the review?**

| Yes  No | For Yes:  There was no significant heterogeneity in the results  OR if heterogeneity was present the authors performed an investigation of sources of any heterogeneity in the results and discussed the impact of this on the results of the review |
| --- | --- |
| **Rating rationale:** | |

**15. If they performed quantitative synthesis did the review authors carry out an adequate investigation of publication bias (small study bias) and discuss its likely impact on the results of the review?**

| Yes  No  No meta-analysis conducted | For Yes:  performed graphical or statistical tests for publication bias and discussed the likelihood and magnitude of impact of publication bias |
| --- | --- |
| **Rating rationale:** | |

**16. Did the review authors report any potential sources of conflict of interest, including any funding they received for conducting the review?**

| Yes  No | For Yes:  The authors reported no competing interests OR  The authors described their funding sources and how they managed potential conflicts of interest |
| --- | --- |
| **Rating rationale:** | |

**To cite this tool:** Shea BJ, Reeves BC, Wells G, Thuku M, Hamel C, Moran J, Moher D, Tugwell P, Welch V, Kristjansson E, Henry DA. AMSTAR 2: a critical appraisal tool for systematic reviews that include randomised or non-randomised studies of healthcare interventions, or both. BMJ. 2017 Sep 21;358:j4008.
