# Supplementary material for: Near-field and far-field exposures to radiofrequency electromagnetic fields and cancer risks in humans: a protocol for an umbrella review of epidemiological studies
Source: Syst Rev. 2026 Mar 12;15:130. doi: 10.1186/s13643-026-03142-9 (PMC13072611; doi:10.1186/s13643-026-03142-9)
Supplement: Supplementary file 4 — Additional file 4: ROBIS: Tool to assess risk of bias in systematic reviews. Description: A Microsoft Word sheet that will be used for applying the critical appraisal criteria of ROBIS. [file 13643_2026_3142_MOESM4_ESM.docx]

**ROBIS: Tool to assess risk of bias in systematic reviews**

| **Study ID** | **Study Reference** | **Assessor** | **Date** |
| --- | --- | --- | --- |
|  |  |  |  |

**INCLUDE PHASE 1?**

**Phase 2: Identifying concerns with the review process**

Y=YES, PY=PROBABLY YES, PN=PROBABLY NO, N=NO, NI=NO INFORMATION

**DOMAIN 1: STUDY ELIGIBILITY CRITERIA**

Describe the study eligibility criteria, any restrictions on eligibility and whether there was evidence that objectives and eligibility criteria were pre-specified:

|  | **Y** | **PY** | **PN** | **N** | **NI** |
| --- | --- | --- | --- | --- | --- |
| 1.1 Did the review adhere to pre-defined objectives and eligibility criteria? |  |  |  |  |  |
| 1.2 Were the eligibility criteria appropriate for the review question? |  |  |  |  |  |
| 1.3 Were eligibility criteria unambiguous? |  |  |  |  |  |
| 1.4 Were any restrictions in eligibility criteria based on study characteristics appropriate (e.g. date, sample size, study quality, outcomes measured)? |  |  |  |  |  |
| 1.5 Were any restrictions in eligibility criteria based on sources of information appropriate (e.g. publication status or format, language, availability of data)? |  |  |  |  |  |

|  | **LOW** | **HIGH** | **UNCLEAR** |
| --- | --- | --- | --- |
| Concerns regarding specification of study eligibility criteria |  |  |  |
| Rationale for concern: | | | |

**DOMAIN 2: IDENTIFICATION AND SELECTION OF STUDIES**

Describe methods of study identification and selection (e.g. number of reviewers involved):

|  | **Y** | **PY** | **PN** | **N** | **NI** |
| --- | --- | --- | --- | --- | --- |
| 2.1 Did the search include an appropriate range of databases/electronic sources for published and unpublished reports? |  |  |  |  |  |
| 2.2 Were methods additional to database searching used to identify relevant reports? |  |  |  |  |  |
| 2.3 Were the terms and structure of the search strategy likely to retrieve as many eligible studies as possible? |  |  |  |  |  |
| 2.4 Were restrictions based on date, publication format, or language appropriate? |  |  |  |  |  |
| 2.5 Were efforts made to minimise error in selection of studies? |  |  |  |  |  |

|  | **LOW** | **HIGH** | **UNCLEAR** |
| --- | --- | --- | --- |
| Concerns regarding methods used to identify and/or select studies |  |  |  |
| Rationale for concern: | | | |

**DOMAIN 3: DATA COLLECTION AND STUDY APPRAISAL**

Describe methods of data collection, what data were extracted from studies or collected through other means, how risk of bias was assessed (e.g. number of reviewers involved) and the tool used to assess risk of bias:

|  | **Y** | **PY** | **PN** | **N** | **NI** |
| --- | --- | --- | --- | --- | --- |
| 3.1 Were efforts made to minimise error in data collection? |  |  |  |  |  |
| 3.2 Were sufficient study characteristics available for both review authors and readers to be able to interpret the results? |  |  |  |  |  |
| 3.3 Were all relevant study results collected for use in the synthesis? |  |  |  |  |  |
| 3.4 Was risk of bias (or methodological quality) formally assessed using appropriate criteria? |  |  |  |  |  |
| 3.5 Were efforts made to minimise error in risk of bias assessment? |  |  |  |  |  |

|  | **LOW** | **HIGH** | **UNCLEAR** |
| --- | --- | --- | --- |
| Concerns regarding methods used to collect data and appraise studies |  |  |  |
| Rationale for concern: | | | |

**DOMAIN 4: SYNTHESIS AND FINDINGS**

Describe synthesis methods:

|  | **Y** | **PY** | **PN** | **N** | **NI** |
| --- | --- | --- | --- | --- | --- |
| 4.1 Did the synthesis include all studies that it should? |  |  |  |  |  |
| 4.2 Were all pre-defined analyses reported or departures explained? |  |  |  |  |  |
| 4.3 Was the synthesis appropriate given the nature and similarity in the research questions, study designs and outcomes across included studies? |  |  |  |  |  |
| 4.4 Was between-study variation (heterogeneity) minimal or addressed in the synthesis? |  |  |  |  |  |
| 4.5 Were the findings robust, e.g. as demonstrated through funnel plot or sensitivity analyses? |  |  |  |  |  |
| 4.6 Were biases in primary studies minimal or addressed in the synthesis? |  |  |  |  |  |

|  | **LOW** | **HIGH** | **UNCLEAR** |
| --- | --- | --- | --- |
| Concerns regarding the synthesis and findings |  |  |  |
| Rationale for concern: | | | |

**Phase 3: Judging risk of bias**

Summarise the concerns identified during the Phase 2 assessment:

| **Domain** | **Concern** | **Rationale for concern** |
| --- | --- | --- |
| 1. Concerns regarding specification of study eligibility criteria |  |  |
| 2. Concerns regarding methods used to identify and/or select studies |  |  |
| 3. Concerns regarding methods used to collect data and appraise studies |  |  |
| 4. Concerns regarding the synthesis and findings |  |  |

**RISK OF BIAS IN THE REVIEW**

Describe whether conclusions were supported by the evidence:

|  | **Y** | **PY** | **PN** | **N** | **NI** |
| --- | --- | --- | --- | --- | --- |
| A. Did the interpretation of findings address all of the concerns identified in Domains 1 to 4? |  |  |  |  |  |
| B. Was the relevance of identified studies to the review's research question appropriately considered? |  |  |  |  |  |
| C. Did the reviewers avoid emphasizing results on the basis of their statistical significance? |  |  |  |  |  |

|  | **LOW** | **HIGH** | **UNCLEAR** |
| --- | --- | --- | --- |
| Risk of bias in the review |  |  |  |
| Rationale for risk: | | | |
